# Recent Advances in Precision Diamond Wire Sawing Monocrystalline Silicon

**DOI:** 10.3390/mi14081512

**Published:** 2023-07-27

**Authors:** Ansheng Li, Shunchang Hu, Yu Zhou, Hongyan Wang, Zhen Zhang, Wuyi Ming

**Affiliations:** 1 Hydropower Equipment and Intelligent System Engineering Technology Research Centre of Henan Province, Department of Mechanical and Electronic Engineering, Henan Vocational College of Water Conservancy and Environment, Zhengzhou 450002, China; liansheng@zzuli.edu.cn; 2 Mechanical and Electrical Engineering Institute, Zhengzhou University of Light Industry, Zhengzhou 450002, China; hushunchang2022@gmail.com; 3 Gokin Solar Company Limited, Zhuhai 519031, China; yu.zhou@gokinsolar.com; 4 Guangdong Provincial Key Laboratory of Digital Manufacturing Equipment, Guangdong HUST Industrial Technology Research Institute, Dongguan 523808, China; hongyanwang923@163.com; 5 School of Aerospace Engineering, Huazhong University of Science and Technology, Wuhan 430074, China

**Keywords:** diamond wire sawing, monocrystalline silicon, precision machining, hybrid machining

## Abstract

Due to the brittleness of silicon, the use of a diamond wire to cut silicon wafers is a critical stage in solar cell manufacturing. In order to improve the production yield of the cutting process, it is necessary to have a thorough understanding of the phenomena relating to the cutting parameters. This research reviews and summarizes the technology for the precision machining of monocrystalline silicon using diamond wire sawing (DWS). Firstly, mathematical models, molecular dynamics (MD), the finite element method (FEM), and other methods used for studying the principle of DWS are compared. Secondly, the equipment used for DWS is reviewed, the influences of the direction and magnitude of the cutting force on the material removal rate (MRR) are analyzed, and the improvement of silicon wafer surface quality through optimizing process parameters is summarized. Thirdly, the principles and processing performances of three assisted machining methods, namely ultrasonic vibration-assisted DWS (UV-DWS), electrical discharge vibration-assisted DWS (ED-DWS), and electrochemical-assisted DWS (EC-DWS), are reviewed separately. Finally, the prospects for the precision machining of monocrystalline silicon using DWS are provided, highlighting its significant potential for future development and improvement.

## 1. Introduction

**Figure 1:**
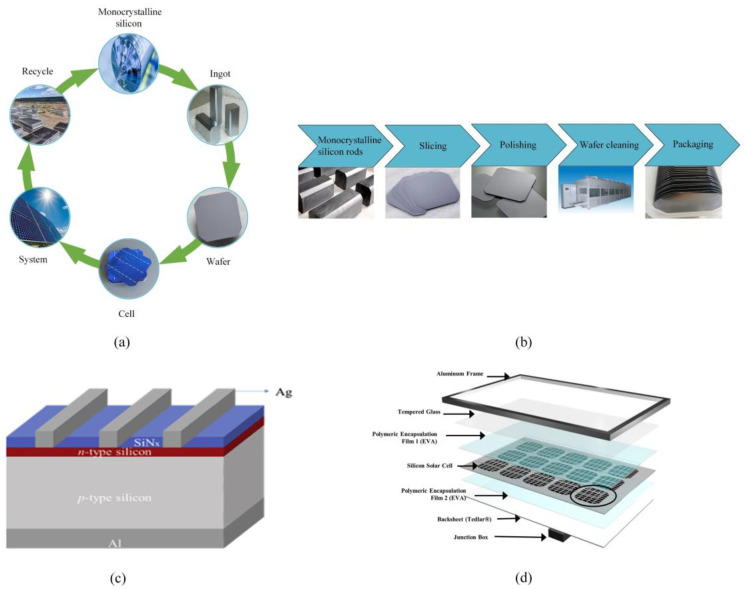
Photovoltaic modules made of silicon. (**a**) A diagram of the whole supply chain of photovoltaic manufacturing; (**b**) a diagram of the silicon wafer production process; (**c**) a schematic diagram of crystalline silicon photovoltaic solar cells [7] and (**d**) a photovoltaic panel’s structure [11].

Currently, solar photovoltaic power is experiencing rapid development due to its advantages, which include utilizing inexhaustible resources, its high degree of reliability, low operational costs, and lack of pollution [1,2,3]. Solar cells are the photoelectric conversion devices used in photovoltaic systems, with silicon being the primary semiconductor material [4,5]. Due to its distinctive physical and chemical properties, monocrystalline silicon has become an essential foundational material in the semiconductor sector [6]. According to reports, over 90% of electronic components are made from silicon materials [7]. Figure 1a shows a diagram of the entire supply chain for the manufacturing of photovoltaic cells. Monocrystalline silicon occupies a prominent role in the global photovoltaic sector due to its well-established material preparation techniques and exceptional photoelectric conversion efficiency [8,9]. Figure 1b shows a schematic of the process of producing silicon wafers.

The solar cell is the essential component responsible for the generation of photovoltaic power using silicon-based photovoltaic modules, which are widely recognized as the most popular photovoltaic technology [7,10]. A standard silicon solar cell consists of a *p*-type or *n*-type doped silicon wafer, approximately 200 µm thick, which acts as the light absorber. Phosphorus or boron, as opposite-polarity dopants, are diffused into the wafer to form a *p-n* junction, generating photovoltage. The current is extracted to the external circuit through Ag, Al, or Cu electrodes. As shown in Figure 1c, the surface is textured and coated with an anti-reflection layer, such as silicon nitride, that is less than 100 nm thick to minimize reflection. To ensure durability under challenging environmental conditions, the solar cells, which are fragile in nature, are integrated into a module structure [7]. The structure of a crystalline silicon photovoltaic module is shown in Figure 1d. The outer layer consists of an aluminum alloy frame, which serves to protect the internal structure. At the bottom, there is a junction box that facilitates the conversion, storage, and transmission of the collected energy. The internal layering, from top to bottom, includes tempered glass, a polymer encapsulation material, silicon solar cells, a polymer encapsulation material, and a backsheet. Typically, ethylene vinyl acetate (EVA) is used as the encapsulation material, aiding in the adhesion of the solar cells to the tempered glass and backsheets [11].

Renowned for its high degree of hardness, brittleness, and excellent chemical stability at room temperature, silicon is a crucial semiconductor material. The techniques of processing silicon include acid etching and wire cutting [12,13]. Acid etching is the chemical etching of silicon materials [14], such as using HNO_3_-HF for isotropic etching [15], employing KOH or NaOH for anisotropic etching [16], and utilizing plasma-etching techniques. However, due to the expensive equipment required and the significant risks posed to operators, chemical methods have not been widely employed in the large-scale production of silicon wafers [17]. The cutting method refers to the cutting of monocrystalline silicon rods into thin slices or wafer shapes [18]. Commonly used cutting methods include wire saw cutting, internal stress cutting, and laser cutting. Wire saw cutting is the most commonly used method in which a monocrystalline silicon rod is placed on a cutting machine and cut into thin slices or wafers via diamond wire sawing (DWS) [19]. The production of silicon wafers typically involves slicing, and the main slicing processes can be categorized into free abrasive wire saw and fixed abrasive DWS techniques. The dominant method of cutting silicon wafers has shifted from free abrasive slurry wire sawing to fixed abrasive DWS [20,21]. The DWS method is effective at cutting monocrystalline silicon material due to the diamond’s high degrees of hardness and sharpness, resulting in high-precision cutting results [22,23]. This method is widely applied in the processes of precision manufacturing semiconductor devices, optical components, and other monocrystalline silicon products [24]. In addition, the thinning process entails the removal of the majority of the monocrystalline silicon wafer’s thickness, leaving only 5% for chip manufacturing [8]. Consequently, reducing the thickness of chip slices becomes a crucial strategy for enhancing the utilization of material and minimizing manufacturing costs [25,26].

**Figure 2:**
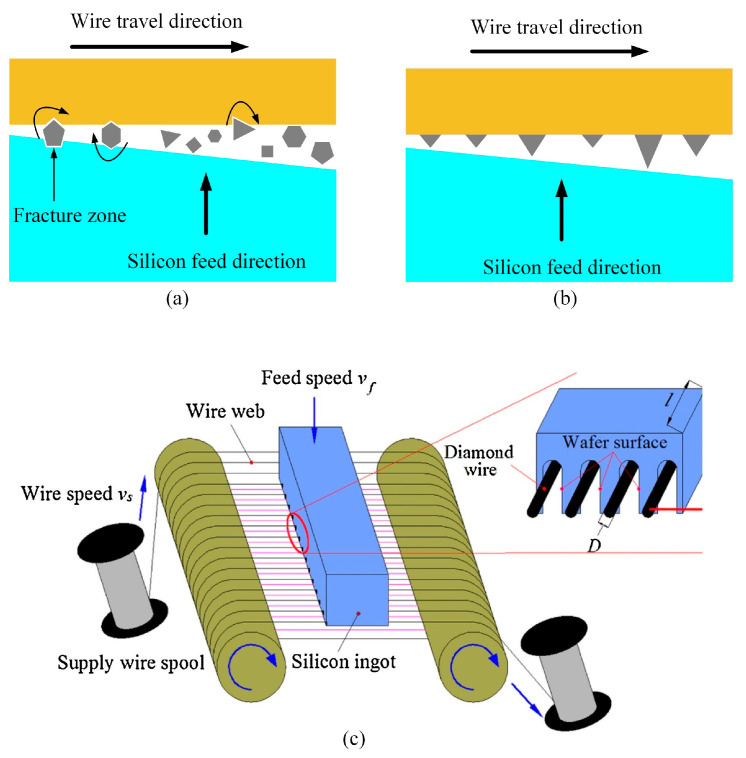
Schematic diagram of the material removal pattern: (**a**) free abrasive cut; (**b**) fixed abrasive cut; (**c**) schematic diagram of cutting silicon ingots with fixed abrasive DWS [27].

DWS is a fixed abrasive cutting technique that involves bonding and electroplating diamond particles onto a stainless-steel wire, enabling rapid movement during the cutting process. Figure 2a illustrates the process of using loose abrasive slurry (LAS) to slice brittle silicon wafers. This process involves the cutting action of the hard, abrasive SiC particles present in a polyethylene glycol (PEG) slurry. The cutting groove contains ingrained sand particles which facilitate the slicing of silicon wafers through a three-body material removal process that occurs between the online cutting grains and the silicon ingot. On the other hand, the fixed abrasive DWS process, as depicted in Figure 2b, resembles the LAS process. The primary distinction lies in the utilization of abrasive particles (diamond) affixed to a steel wire coated with electroplated nickel. Additionally, the PEG slurry is substituted with a water-based cutting fluid. Unlike the three-body material removal mode used in mortar steel wire cutting, DWS utilizes a two-body material removal mode between the diamond particles fixed on the steel wire and the material being cut. The abrasive grains do not roll, and the cutting efficiency can reach several times that of the three-body material removal mode. A schematic diagram of the DWS process is shown in Figure 2c [27]. The contact length between the silicon ingot and the diamond wire is defined as “*l*”, while the diameter of the diamond wire is denoted as “*D*”. The contact length “*l*” corresponds exactly to the length of the silicon ingot. In this process, the diamond wire moves axially at “*v_s_*”, while the silicon ingot is fed perpendicular to the diamond wire grid at “*v_f_*”. Xiao et al. [27] modified and simplified the model.

This paper provides a review of the precision machining of monocrystalline silicon using DWS and analyzes the mechanisms of material removal and surface generation. The Section 2 examines the research employed in DWS processing, covering subjects that range from simulating atomic-scale behavior to handling complex macroscopic behavior. These methods complement each other, offering a comprehensive understanding and prediction of DWS research. The Section 3 reviews the DWS processing procedure, while the Section 4 explores auxiliary methods for precision machining using DWS. In the Section 5, outlooks for the precision machining of monocrystalline silicon using DWS are presented. This research paper aims to enhance comprehension of the impact of process parameters on the mechanisms of material removal and surface generation during the DWS of monocrystalline silicon. It provides significant theoretical and experimental value in comprehending this process, further promoting the development of high-speed precision slicing technology for silicon crystals.

## 2. Models and Simulation for DWS

### 2.1. Mathematical Model

**Figure 3:**
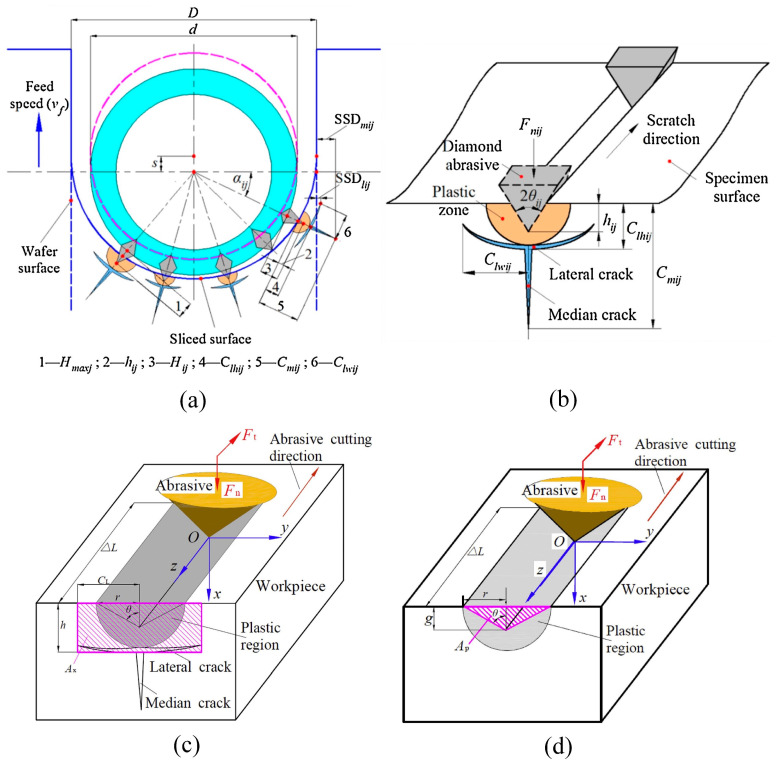
Crack systems for DWS: (**a**) crack system on any wire cross-section [27]; (**b**) crack system on the surface of a brittle specimen scuffed by any abrasive [27]; (**c**) material brittleness removal; (**d**) material ductility removal [39].

DWS technology has become a significant field in modern industry and scientific research [28]. This technique utilizes diamonds as cutting tools and achieves precise cutting and processing on various materials through the high-speed rotation and grinding action of the diamond wire [29]. Chung et al. [30,31,32] proposed a modeling method for DWS, establishing a mathematical analysis model for the sawing process and analyzing the mechanism of sawing surface generation. Due to the random distribution of the diamond abrasives, Xiao et al. [27] introduced a novel model to evaluate the subsurface damage (SSD) depth incurred by solar silicon wafers during the DWS process. The wire sawing technique applied to silicon ingots can be regarded as a scratching process employing diamond abrasives. Figure 3a illustrates the crack system on an arbitrary (or *j*th) cross-section of a wire. In the context of abrasive randomness, it is noteworthy that certain abrasives might not make contact with the surface undergoing cutting. Additionally, some abrasives might execute the wafer cutting process in a ductile manner, thereby avoiding the formation of cracks. However, others may induce lateral cracks and median cracks via brittle cutting modes. This relationship between the abrasive penetration depth *h_ij_* and the critical depth *h_c_* serves as a primary factor in determining the cutting state, as proposed by Nakamura et al. [33]. When 0 < *h_ij_* ≤ *h_c_*, the state is in a ductile mode, while when *h* > *h_c_*, the state is in a brittle mode. Bifano et al. [34] derived an equation for the critical depth *h_c_*, as follows:

(1) $$h_{c}=0.15\frac{E}{H_{S}}\left(\frac{K_{C}}{H_{S}}\right)$$

where *E* is the elastic modulus, *H_s_* is the hardness, and *K_c_* is the fracture toughness of the specimen. Drawing on micro-indentation mechanics, Lawn and Wilshaw [35] developed a representative crack system for the interaction between an abrasive material and a brittle sample surface. Figure 3b depicts this system. Notably, plastic zones form beneath the surface of the specimen, from which both median and lateral cracks originate. Median cracks typically propagate perpendicular to the sample surface, while lateral cracks generally propagate parallel to it. Marshall et al. [36] derived formulas for determining the depth and width of a lateral crack, which can be expressed as follows:

(2) $$C_{lhij}=k{\left(\cot\theta_{ij}\right)}^{1/3}\frac{E^{1/2}}{H_{s}}F_{{}_{nij}}^{{}^{1/2}}$$

(3) $$C_{lwij}=k{\left(\cot\theta_{ij}\right)}^{5/12}{\left(\frac{E^{3/4}}{H_{s}K_{S}{\left(1-v^{2}\right)}^{1/2}}\right)}^{1/2}F_{{}_{nij}}^{{}^{5/8}}$$

The equation for the normal load *F_nij_* of any given abrasive can be expressed as follows, in which *C_lhij_* represents the depth of a lateral crack, *C_lwij_* denotes the width of the lateral crack, *k* is a dimensionless constant (*k* = 0.226) [36], and *ν* represents the Poisson’s ratio value of the specimen. The equation for the normal load *F_nij_* was derived by Williams as follows [37]:

(4) $$F_{nij}=0.5\pi H_{s}h_{ij}^{2}{\left(\tan\theta_{ij}\right)}^{2}$$

For an arbitrary abrasive, the equation describing the depth of a median crack *C_mij_* is as follows:

(5) $$C_{mij}={\left[\left(1+\frac{X_{e}}{X_{r}}\right)\times {\left(\cot\theta_{ij}\right)}^{2/3}{\left(\frac{E}{H_{s}}\right)}^{{}^{1/2}}\frac{F_{nij}}{K_{c}}\right]}^{2/3}$$

This equation incorporates the indentation coefficients *X_e_* for the elastic stress field and the plastic stress field *X_r_* beneath the contact impression, which were determined to be 0.032 and 0.026, respectively [38].

The removal of material brittleness fracture primarily occurs due to the extension of lateral cracks that are almost parallel to the sawing surface, followed by their migration towards the sawing surface, resulting in brittle chips. The expansion of lateral cracks leads to the removal of the material beneath the abrasive, approximately in the form of rectangular blocks. During the process of wire sawing silicon crystals, the cutting action of the abrasive on the wire surface exhibits similarities to the movement of a platen subjected to tangential and normal forces, as depicted in Figure 3c [39]. In this scenario, the volume of material removed is approximated as a cube. The volume of the cube is determined by multiplying the length of the abrasive by the depth of the lateral cracks *h* and the length *C*_L_. When material removal takes place in a ductile mode, it occurs through the shearing action of the abrasive. In this case, the average cutting depth of the abrasive corresponds to the undeformed chip thickness, represented as *g*, as shown in Figure 3d [39] In such cases, the volume of the removed material can be approximated as a triangular prism. To calculate this volume, multiply the height by the area of the triangle. As depicted in Figure 3c, on a cross-section of a processed material, an area *A*_x_ removed by abrasive cutting in a brittle fracture mode can be estimated as follows [39]:

(6) $$A_{x}=2\cdot C_{L}\cdot h$$

A theoretical formula exists for calculating the depth *h* of lateral cracks, which can be expressed as follows [40]:

(7) $$h=0.43{\left(\sin\theta \right)}^{1/2}{\left(\cot\theta \right)}^{1/3}{\left(\frac{E}{H}\right)}^{1/2}{\left(\frac{F_{n}}{H}\right)}^{1/2}$$

The lateral crack generation depth (in μm), denoted by *h*, and Young’s modulus (in GPa), represented by *E*, are key factors in the equation. *F*_n_ corresponds to the normal force exerted on a single abrasive (in N), while *θ* signifies the half-angle (in °) of the abrasive edge.

Mathematically expressed, the length of lateral crack propagation, denoted as *C*_L_, can be determined using the following Equation [41]:

(8) $$C_{L}=\alpha \frac{E^{3/8}}{{\left(K_{\mathrm{IC}}\cdot H\right)}^{1/2}}F_{n}^{5/8}$$

The material fracture toughness, denoted as *K*_IC_ and measured in MPa·m^1/2^, and *α*, which represents the indentation constant associated with the shape of the abrasive, are important parameters in the equation. For a Vickers indenter with an angle of 140°, the value of *α* is 0.12.

As illustrated in Figure 3d, when considering the cutting direction of the abrasive, the area removed *A_p_* during ductile-shear-mode abrasive cutting can be approximately calculated using the following formula [39]:

(9) $$A_{P}=gr$$

If the abrasive cuts a length Δ*L*, the volume of material removed in the ductile mode, denoted as *V_sigd_*, can be represented by the following equation [39]:

(10) $$V_{sigd}=\Delta L\cdot A_{P}=\Delta L\cdot gr$$

By conducting research on the nucleation position and propagation direction of cracks in abrasive cutting, Li et al. [42] formulated a sawing model that incorporates both ductile and brittle material removal in DWS. This model was utilized to investigate the impacts of process parameters on cutting depth and surface roughness during the cutting of monocrystalline silicon. Wu et al. [43] analyzed the distribution and size range of surface cracks in the DWS of silicon wafers via experimental statistics and simulations. The results showed that the cracks at the wafer edge had greater depths than those at the center, and the crack sizes approximately followed a Weibull distribution. The critical speed ratio between the feed rate and traverse speed in the DWS of monocrystalline silicon was examined by Yin et al. [44], who also explored the sawing process parameters that promote ductile material removal on the wafer surface. In fact, the fracture strength of the cut silicon wafer was influenced not only by the depth of the subsurface cracks but also by their distribution characteristics, such as the number and size of the cracks. Furthermore, it influences the resulting surface etching texture of the wafer.

### 2.2. Finite Element Methods

The finite element method (FEM) is a numerical analysis method used to solve mathematical models of real engineering problems which divides complex continuous media into small discrete elements and establishes mathematical equations within each element. By solving these equations, it is possible to predict and analyze the behavior and performance of materials, structures, or systems [45]. The FEM discretizes the geometry and physical properties of a structure or system into a finite number of elements. Each element has its own mathematical description and forms a discrete mathematical model of the entire structure through the connectivity of nodes. The interactions between these elements and the external loading conditions are defined by boundary conditions and material properties [46]. By solving the system of equations in the model, the response of the structure or system, such as displacements, stresses, strains, and other relevant physical quantities, can be obtained [47]. FEM simulations are widely used in various fields, including structural mechanics, heat conduction, fluid mechanics, electromagnetics, and multi-physics coupling problems [48,49,50,51]. FEM provides powerful tools for engineering design, material analysis, performance optimization, and fault diagnosis.

**Figure 4:**
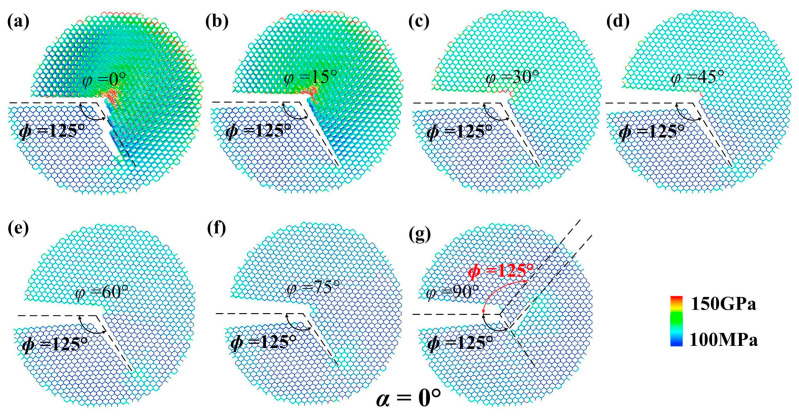
The crack growth angles of {110} monocrystalline silicon plates for a given chiral angle α = 0° and t = 17.28 Å under different loading angles using the FEM method: (**a**) φ = 0°; (**b**) φ = 15°; (**c**) φ = 30°; (**d**) φ = 45°; (**e**) φ = 60°; (**f**) φ = 75°; (**g**) φ = 90° [52].

Using MD simulations and FEM calculations based on the boundary layer model, Wei et al. [52] derived critical stress strength factors and crack propagation angles for the mixed mode in monocrystalline silicon plates with {100} and {110} orientations. The FEM calculations in Figure 4 illustrate the crack expansion angles for various chiral angles (t = 17.28 Å for the {110} plate) at different loading angles. In Figure 4a–g, the FEM calculations demonstrate that *ϕ* reaches 125° ({110} plates of t = 17.28 Å) as the loading angle φ changes from 0° to 90°, while in Figure 4g, *ϕ* initially equals 125° and then transitions to −125°, suggesting a tendency for *ϕ* to approach 180°. Extensive investigations were conducted by Zhang et al. [53] on the anisotropic elasticity of silicon, utilizing matrix-based computational algorithms. Their study established a correlation between thermal deformation and Poisson’s ratio. Furthermore, Hauch et al. [54] performed experiments to measure the velocity of running cracks in brittle monocrystalline silicon, studying their relationship with crack tip energy flux. Their experimental findings were validated through direct comparisons with MD simulations based on an enhanced Stillinger-Weber (SW) interatomic potential. Zhu et al. [55] made significant progress by providing the first atomic-scale determination of the minimum energy path for crack propagation, which was achieved by breaking bonds along the crack front. Additionally, Cook [56] focused on investigating the strength and brittle fracture behavior of silicon under sharp contact points, shedding light on the fracture characteristics of silicon with different contact defects. 

The residual microcracks left on the surfaces and subsurfaces of silicon wafers due to brittle material removal are prone to expansion and interference under the stress of sawing, leading to a decrease in fracture strength and resulting in fracture. Nevertheless, experimentally measuring the dynamic variation and distribution of sawing stress remains challenging. Cheng et al. [57] used the FEM to model multi-wire sawing on polycrystalline silicon based on a cutting force model for DWS. The study investigated the distribution and coupling characteristics of sawing stress and the influences of process parameters and silicon wafer size on stress values and distribution. The research findings demonstrate that the values of mechanical stress during the sawing process were relatively small and contributed less to the coupled stress; however, they did affect the distribution of the coupled stress, with thermal stress playing a dominant role. Wallburg et al. [58] conducted an experimental study on the removal coefficient using FEM simulation calculations. The results revealed a significant increase in material removal under DWS conditions compared to loose abrasive sawing (LAS). In the field of study of temperature during cutting, Bhagavat et al. [59] developed a two-dimensional FEM model to analyze the variations in temperature while slicing a silicon wafer using a wire sawing technique. They investigated the heat flux and convective heat transfer boundary conditions during the slicing process and controlled the temperature variations to reduce wafer warpage. Gao et al. [60] employed the FEM to simulate the maximum principal stress and depth of subsurface microcrack damage during the DWS of SiC. Wu et al. [61] conducted an FEM analysis on diamond abrasive multi-wire saw silicon rods. Based on the removal mechanism of brittle materials, when the maximum cutting depth of a monocrystalline silicon rod was smaller than the critical cutting thickness, the cutting mode of the multi-wire saw silicon rod was characterized as plastic removal. A comparison between the deformation obtained from the FEM analysis and the maximum cutting depth revealed an error of less than 15%.

### 2.3. Molecular Dynamics Model

Molecular dynamics (MD) is a computational method used to simulate the temporal evolution of atoms and molecules [62,63,64]. It is based on the principles of classical mechanics and utilizes Newton’s laws of motion and Coulomb’s law to describe the interactions between particles [65,66,67]. By simulating the motion of atoms and molecules under various conditions such as temperature, pressure, and volume, molecular MD enables the exploration of the properties and reaction processes of materials [68]. MD simulation methods can be employed to determine the positions and velocity components of all atoms in a system at any given time during the simulation. This enables the calculation of microscopic mechanistic information, such as dynamic and thermodynamic properties, within the material. By predicting and guiding the research on and production of novel materials, these findings help save significant resources and time in terms of manpower and material costs [69,70]. As a result, it finds wide-ranging applications in fields such as material science, biochemistry, and chemical engineering [71,72,73].

The mechanical properties of silicon have been extensively studied, with particular interest paid to understanding the impact of the applied strain rate on silicon’s mechanical performance, fracture behavior, and deformation mechanisms [74,75]. Investigating these aspects not only enhances our comprehension of damage tolerance and deformation behavior [76] but also opens up new possibilities and challenges for silicon nanodevices and silicon-based anodes [77]. Typically, three commonly used methods are employed to investigate the mechanical response of silicon under varying strain rates. The first approach entails theoretical studies that consolidate and forecast the rate-dependent mechanical responses, encompassing constitutive equations [78], models of dislocation plasticity [79,80,81], and response models that consider rate dependency [82]. However, the intricate material characteristics of silicon, including the well-known transition from brittleness to ductility, often introduce complexity into theoretical investigations. The second approach involves nano-mechanical experiments conducted at low strain rates [83,84] (typically <100/s). Nevertheless, certain conditions, such as defect-free and extremely high strain rates, are challenging to replicate in experiments. Hence, researchers frequently employ the third approach: high-strain-rate MD simulations [85,86] (typically >108/s). Recent simulations have revealed that while the plasticity of brittle, glassy nanowires remains unchanged with a decreasing strain rate, the plasticity of their ductile counterparts decreases [87].

**Figure 5:**
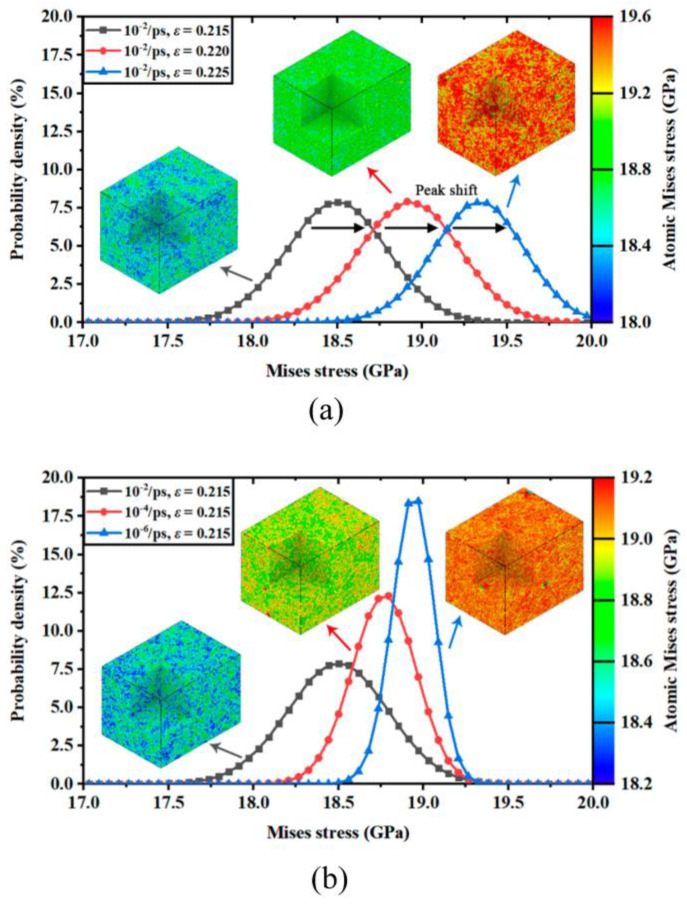
The probability density of von Mises stress distribution in monocrystalline silicon [74]: (**a**) under three different applied strains and the same strain rate; (**b**) under three different strain rates and the same applied strain.

Liu et al. [74] employed a rate-response model to determine the strain rate sensitivities and critical strain rates for two different structures. They conducted a comparative atomic analysis of perfect monocrystalline silicon crystals and silicon nanowires to investigate the impact of strain rate on the mechanical behavior of these structures. The study also examined the rate-dependent dislocation activity during fracture. The analysis and discussion included an examination of the effect of strain rate on the equivalent stress and corresponding yield criteria for the two silicon structures. However, the influence of strain rate on the von Mises stress during the deformation process remains uncertain. In order to explore this aspect more comprehensively, the distribution of the von Mises stress is presented in the form of probability density in Figure 5a (monocrystalline) and Figure 5b (silicon nanowire). It is evident that the numerical distribution of the von Mises stress follows a normal distribution pattern. With variations in the applied strain, the stress peak gradually shifts towards higher values, as depicted in Figure 5a. Interestingly, the shape of the stress peak differs at different strain rates. In high-strain-rate scenarios, the atomic virial stress appears to be spread across a wider range. Conversely, the width of the distribution peak narrows during deformation at lower strain rates. By comparing the stress distributions at three specified strain rates under the same applied strain in Figure 5b, it is observed that the peak position (average stress) varies.

Based on previous research on nanoscratching and nanoidentation models, Liu [88] proposed and developed an innovative MD model for DWS. An MD simulation was performed to analyze the diamond wire cutting process on a monocrystalline silicon workpiece using a tool grain with a radius of 2.142 nm. In the ultra-precision diamond wire cutting process, the removal of the monocrystalline silicon material primarily follows a ductile removal mode. The bottom of the diamond grain mainly interacts with the silicon atoms of the monocrystalline workpiece, primarily through extrusion. Alternatively, the side of the grain primarily contributes to the formation of the final machined surface, typically with very low cutting depths, and the dominant modes are scratching and plowing. Chung and Le [32] developed a model for DWS and analyzed the cutting depths at different positions on the silicon wafer surface for various process parameters. Furthermore, increasing the cutting wire speed and feed rate to a certain extent can improve the surface quality of silicon wafers.

### 2.4. Summary

Mathematical modeling methods involve establishing mathematical equations and models to derive key parameters and behaviors of DWS. These models are typically based on the principles of mechanics, thermodynamics, and material properties, providing a quantitative description and prediction of the cutting process. The advantage of mathematical models is that they offer concise analytical results and serve as theoretical foundations. However, in complex situations, they may rely on certain assumptions and simplifications. FEM simulation is a numerical method based on the principles of continuum mechanics and is used to simulate the macroscopic behavior of DWS. It discretizes the cutting tool, workpiece, and interfaces into a finite number of elements and solves the corresponding mechanical equations and boundary conditions to obtain the stress, deformation, and cutting forces. An FEM analysis excels in handling complex geometric shapes and boundary conditions, providing detailed stress and deformation distributions. However, accurately modeling material properties and cutting process details requires appropriate material and interface models. MD simulation utilizes atomistic-scale modeling techniques to study the microscopic behavior of DWS by simulating the interactions and movements of atoms. It provides detailed descriptions of atomic positions, velocities, stresses, and deformations, revealing atomic-scale mechanisms during the cutting process. The advantage of MD simulation is its ability to consider the real atomic structure and complexity of a material. However, it requires significant computational resources and has limitations in terms of system size and time scale.

The mathematical analysis model is a theoretical and mathematical approach that allows for the direct validation of specific problems or system solutions. FEM allows for the direct validation of experimental results by comparing simulated outcomes with actual experimental data to evaluate and verify the accuracy and reliability of numerical models. The MD model is a computational simulation method used to study the motion and interactions of atoms and molecules. While MD models provide detailed information at the atomic level and are significant for investigating material micro-mechanisms and interactions, they often entail high computational costs, requiring substantial computational resources and time. Therefore, MD models are predominantly utilized for fundamental scientific inquiries and specific research domains to indirectly validate and explain experimental results.

The mathematical analysis model requires the establishment of accurate equations, formulas, and models, which can be challenging to achieve for complex systems or nonlinear problems. FEM discretizes continuous physical systems into a finite number of subdomains (elements), making discretization errors inevitable, especially when the number of elements is small or chosen improperly, which can hinder simulation capabilities. MD models operate at the atomic and molecular scales and are thus limited by time and spatial scales. For larger-scale systems or longer simulations, the computational costs significantly increase. Table 1 summarizes the DWS precision machining models and simulation types.

For the analysis of sawing conditions, FEM can provide accurate stress and deformation distributions, aiding in achieving an understanding of the material’s behavior during the sawing process. However, FEM also has its limitations, particularly when it comes to handling atomic-level details and precise molecular motions. This is where the advantages of the MD model come into play. The MD model can simulate the interactions between atoms and molecules, and in the study of sawing conditions, it can help reveal the material behavior at the atomic level and the mechanisms of crack propagation. Therefore, combining FEM and MD models allows for a more comprehensive and in-depth understanding. Lee and Basaran [92] proposed a novel multiscale modeling technique that combines MD and FEM. This method, which relies on the principle of weighted average momentum, offers a synergistic integration of MD and FEM. MD and FEM are particularly well-suited for achieving specific levels of accuracy in atomistic simulations and continuum simulations, respectively. By employing FEM for macroscopic-scale modeling and analysis while simultaneously utilizing the MD model to study microscopic-scale details, one can better explore the behavior and performance of materials under sawing conditions. This integrated approach provides insights across scales, facilitating a comprehensive understanding of the sawing process and offering more accurate guidance for material design and optimization. Taking into account the brittleness of silicon, the effectiveness of each these methods was compared and summarized. The analytical approach cannot directly simulate cracks but can provide detailed stress field information, which aids in predicting crack initiation. Moulins et al. [93] employed quantitative fracture techniques to perform dynamic crack modeling and stress field analysis in monocrystalline silicon. By comparing the predicted fracture characteristics from the analytical model with experimental observations, they established the dynamic crack propagation behavior within the silicon and determined the asymptotic stress field at the crack tip. An FEM model can simulate cracks, but it is not capable of large-scale simulations yet as novel methods like the extended finite element method (XFEM) are still underdeveloped. Cervera et al. [94] critically compared three modeling approaches for predicting the failure of quasi-brittle structures and examined the relative performances of XFEM, mixed strain/displacement FEM, and phase-field modeling. Through the comparison, it was observed that the cost of XFEM far outweighed its benefits. Mixed strain/displacement FEM emerged as a versatile framework for addressing mechanical problems, improving the accuracy of the discretized strain field and introducing the necessary regularization for handling a discrete-level fracture. The phase-field model was considered a regularization of linear fracture problems at a continuous level, and all three methods shared common drawbacks in discontinuous crack approaches. On the other hand, MD can simulate cracks more realistically compared to analytical approaches or FEM approaches, but its scale is extremely limited [89,95]. 

In summary, mathematical models offer theoretical foundations and concise analytical solutions: MD simulations consider atomic-scale micro-behavior, and FEM analyses can simulate macroscopic behavior and handle complex situations. These methods complement each other in the study of DWS, providing comprehensive understanding and predictive capability. 

## 3. Machining Performance of DWS

### 3.1. DWS Equipment

As mentioned above, DWS plays a key role in the manufacturing processes of solar cells [4]. Thinning silicon wafers have gained significant popularity in the semiconductor industry, becoming a prevalent trend. By reducing the thickness of silicon wafers, material utilization can be improved, and manufacturing costs can be lowered [96]. In the manufacturing of solar cells, DWS is utilized to cut silicon wafers into thinner crystalline slices [97]. This thinning process contributes to enhancing the efficiency and performance of photovoltaic cells. Thinner silicon wafers exhibit superior light absorption and photovoltaic conversion characteristics, enabling a more efficient conversion of solar energy into electricity [98]. Additionally, thin silicon wafers possess lower masses and reduced thermal losses, thereby improving the stability and reliability of solar cells [99]. Through DWS, silicon wafers can be cut into extremely thin chips, allowing for greater integration density and increased functionality. Moreover, reducing the thickness of silicon wafers aids in lowering wafer costs and enhances chip yield and performance [100]. DWS, as an efficient and precise cutting technique, provides a critical solution for the manufacturing of solar cells. It not only enables the thinning of silicon wafers but also ensures high-quality cutting surfaces and minimal cutting losses [1]. As the demand for more efficient, compact, and cost-effective devices continues to grow, further advancements and improvements in DWS technology will play a significant role in solar energy [101,102].

**Figure 6:**
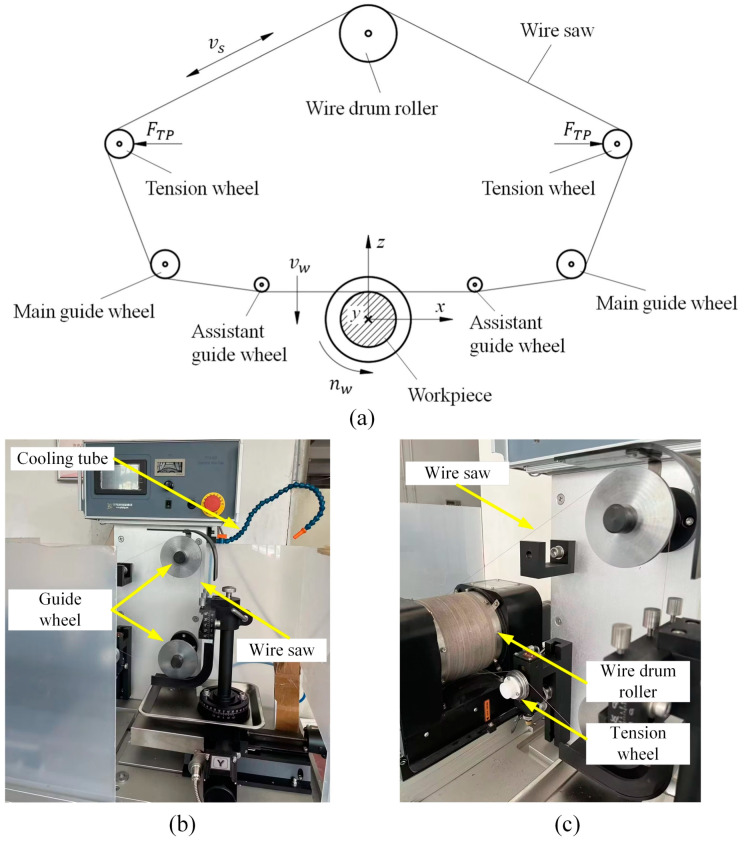
DWS processing and manufacturing process: (**a**) schematic diagram of DWS [1]; (**b**,**c**) DWS equipment.

A schematic diagram of DWS equipment is shown in Figure 6a. The wire saw is wrapped around a drum, two tensioning wheels, two guiding wheels, and two auxiliary guiding wheels, forming a loop. The workpiece is a cylindrical monocrystalline silicon rod. A machine coordinate system, denoted as *oxyz*, is established with the center of the cutting section of the workpiece as the origin, with the x-axis pointing horizontally to the right, the z-axis pointing vertically upward, and the y-axis pointing inward, perpendicular to the paper. During the machining process, a constant tension force *F_TP_* is applied to tighten the wire saw through the use of cylinders and tensioning wheels. The drum roller is rotated by the motor, causing the wire saw to execute back-and-forth movements with a linear velocity of *v*_s_. Meanwhile, the drum roller, tensioning wheels, guiding wheels, and auxiliary guiding wheels rotate. The same machine frame drives the wheel to feed vertically downward with a feed rate of *v_w_*. At a rotational speed of *n_w_*, the spindle drives the workpiece to execute consistent circular motion along the y-axis. 

Teomete et al. [103] conducted silicon sawing experiments using diamond wires with different parameters under the same process conditions. The study revealed that the performance of the diamond wires significantly impacted the surface roughness. Reducing the spacing between abrasives on the wire surface while maintaining a certain chip clearance space contributed to enhancing the quality of the sawn surface. In an experimental study on monocrystalline silicon DWS, Suzuki et al. [104] explored different process conditions. The experiments showed that using smaller diamond abrasives reduced surface scratches and micro-pits, leading to improvements in the post-cut surface roughness of the silicon wafers. This positive effect facilitated ductile material removal during the cutting process, enhancing the material’s machinability. Wallburg et al. [105] conducted scratch experiments and a numerical analysis on monocrystalline silicon wafers using Vickers indenters. The research findings indicate that the distance between abrasives influences processing characteristics and the propagation interference of subsurface microcracks. The differences in the distances between abrasives are reflected in variations in the density of the diamond abrasive on the wire surface. A study conducted by Chou et al. [106] demonstrates that increasing the size of the diamond abrasives leads to a deterioration in chip quality and surface microstructure while improving cutting performance. Figure 6b,c show a schematic diagram of the equipment used in the DWS process. In Table 2, the influence of the parameters on the stability of the sawing process is summarized.

The applied tension in the sawing process affects the stability and quality of the cut. Higher tension levels can improve the stability of the sawing process, resulting in reduced vibrations and an improved surface finish. However, excessive tension may increase the sawing force and lead to an increase in surface roughness [107]. Costa et al. [108] identified a brittle transition by detecting the presence of surface residual phases. The findings demonstrate that higher feed rates result in deeper, wider craters and increased surface roughness. As the feed rate increases, the dominance of the brittle mode becomes prominent, while higher cutting speeds lead to the formation of more ductile regions and improvements in roughness. Higher Sa values are observed with an increased feed rate and tension, while Sa decreases with an increase in wire cutting speed. The dominance of the brittle mode becomes prominent as the feed rate increases. Regarding the sawing surface roughness, a longer reciprocating period tends to result in improved surface quality. This is primarily because a longer period allows for smoother and more continuous cutting motion, reducing the occurrence of vibrations and irregularities on the surface [109]. In terms of sawing force, the reciprocating period also plays a role. Generally, a longer reciprocating period tends to decrease the sawing force. This is due to the smoother cutting action enabled by a longer period, which reduces the impact and resistance encountered during the sawing process [110]. Additionally, the reciprocating period has implications for the stability of the sawing process. A longer period helps to enhance the stability of the sawing process by reducing vibrations and potential instabilities [111]. 

In a single-wire DWS, a single wire with diamond particles embedded in it is used for cutting. Additionally, industrial-grade multi-wire DWS utilizes multiple wires with diamond particles to perform cutting simultaneously. The use of multiple wires in the industrial grade setup allows for higher cutting efficiency and increased productivity compared to single-wire saws [112]. Costa et al. [113] conducted experiments using a single diamond wire in a circular configuration to investigate the influence of DWS on the feed force and silicon wafer quality. The results revealed that cutting conditions with higher feed rates and higher levels of wire tension increased the feed force by 78% and 20%, respectively. However, when the cutting speed was increased to enhance the wire cutting rate, the feed force decreased by 66% due to a reduction in the depth of grain penetration. To accommodate larger-diameter wafers, designers have enhanced traditional multi-wire saws by incorporating a swinging structure in the fixed processing rollers. This results in wire oscillation during the cutting process. Chen and Gupta [114] developed a mathematical model for wire oscillation during the wire sawing process, and the results showed that the oscillation mode reduced the contact length by nearly half compared to the case without oscillation at any given time point. With reduced contact between the wire and the ingot, there is an increase in the force per unit abrasive, resulting in more indentations and less sliding, making it easier to cut harder and larger diameter materials. In multi-wire sawing, reducing wire wear and managing wire consumption are common approaches to mitigating the risk of wire breakage, as worn wires undergo a complex process of strength degradation and surface damage. Li et al. [115] investigated the surface morphology and fracture strength of worn wires to study the wear loss of different steel wires. Based on the experimental results, they further proposed a model for the maximum allowable wear limit of the wire, ensuring its safe usage during the sawing process. Anspach et al. [116] introduced structured wires into the mass production of silicon wafers via multi-wire cutting techniques, resulting in a 106% increase in the working table speed. Consequently, wire consumption was reduced by 45%. The structure of the cutting wire varies, but the apparent “outer” diameter of the structured wire decreases. This reduction can lead to an approximate 2 mm decrease in average roller spacing as there are losses in structure and diameter from the first groove to the last. Groove measurements indicate that the reduction in roughness for the structured wire experiments along the grooves is smaller compared to the straight-line experiments. Hence, the use of structured wire can be expected to achieve a more uniform roughness on the wafer surface.

DWS is a cutting process used for the fabrication of silicon wafers. Monocrystalline silicon and polycrystalline silicon are two common types of silicon wafers, and they exhibit distinct differences in their structure and properties, which consequently lead to variations during DWS processing. Due to its continuous crystal structure, monocrystalline silicon is relatively easy to cut during the DWS process, resulting in a small kerf width, which leads to a smoother cutting surface and reduces the likelihood of cracking [1,117]. Extensive theoretical analyses and experimental studies have demonstrated that reducing the ratio of feed rate to wire speed can decrease the surface crack damage of monocrystalline silicon wafers [27,118,119,120]. Due to the discontinuity in its crystal structure, polycrystalline silicon presents greater difficulty in DWS cutting, often necessitating higher cutting forces and resulting in relatively rough cut surfaces, with an increased risk of diamond wire breakage [121]. Meinel et al. [122] discovered that the DWS cutting of polycrystalline silicon wafers leads to the formation of defect bands on the surface, which are located in the troughs of the cutting marks and exhibit anisotropic characteristics. Kumar et al. [123], through an observation of the surfaces of silicon wafers processed via electroplated DWS, found that the geometric appearance of the diamond grains also had a significant impact on the subsurface microcrack damage of the generated silicon wafers. They also compared the cutting effects on monocrystalline silicon and polycrystalline silicon, discovering that when other processing parameters remained unchanged, the degree of damage to monocrystalline silicon wafers was much lower than the degree of damage to polycrystalline silicon wafers.

### 3.2. Material Removal Rate

The cutting force in DWS has a significant impact on the MRR [124]. The magnitude and direction of the cutting force directly influence the friction and cutting action between diamond particles and the workpiece surface. The contact force between the workpiece surface and the diamond particles can be increased by applying a higher cutting force, promoting the cutting action and thereby improving the MRR [125]. However, excessive cutting force can lead to problems. When the cutting force exceeds a certain threshold, it can cause excessive deformation, fracturing, or damage to the workpiece [126,127]. Additionally, the use of an excessive cutting force can accelerate the wear of the diamond wire, reducing the cutting efficiency and life of the tool. During the DWS process, it is essential to control the magnitude of the cutting force while maintaining sufficient force to enhance the MRR. This approach helps prevent adverse effects on both the workpiece and the cutting tool. This can be achieved by optimizing the cutting parameters, selecting appropriate diamond wires, and using suitable cutting conditions [128,129].

During the machining process, various cutting parameters undergo frequent changes. These parameters include the path length (cutting distance), cutting depth, spindle speed/wire speed, and feed rate [130,131]. However, it is widely believed that an increase in the cutting speed leads to an increase in the hydrostatic pressure, resulting in more pronounced phase transformations within the workpiece and the material in front of the cutting tool [132,133]. Larger cutting depths and feed rates accelerate the wear of diamond tools, while an increase in the traverse speed reduces the wear of diamond tools [113,134,135]. The cutting parameters and tool geometry determine the MRR and also influence the occurrence of phase transformations during silicon cutting. Therefore, under high-MRR conditions and a negative rake angle, the occurrences of phase transformations appear to be enhanced. Additionally, the temperature at the tool edge increases, promoting the carbonization and graphitization reactions of diamond, fundamentally increasing the wear on the tool [136,137].

**Figure 7:**
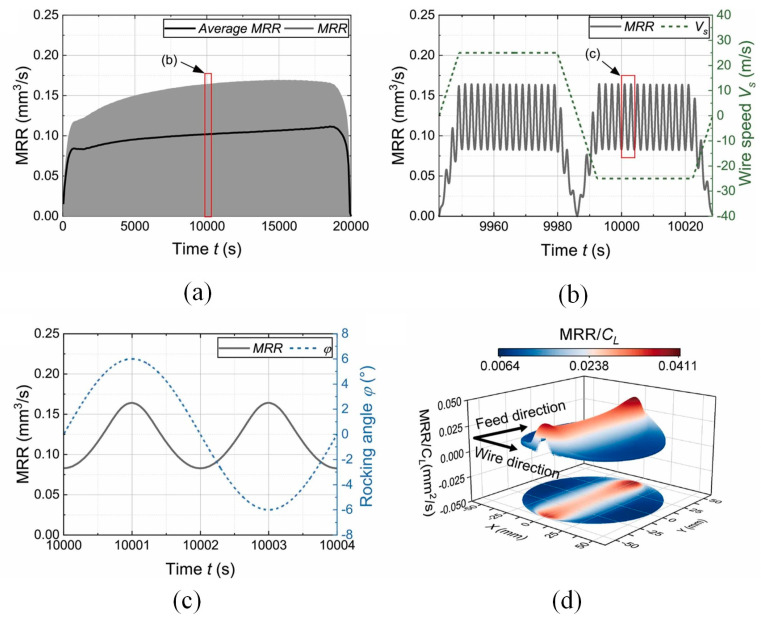
The time curve of MRR and the mapping of MRR per unit contact length [110]; (**a**) variation in MRR throughout the entire sawing process (the red box refers to the effect at 10,000 s in (**b**)); (**b**) influence of wire reciprocating motion on MRR (the red box refers to the effect of the workpiece oscillation on MRR in (**c**)); (**c**) impact of workpiece oscillation on MRR; (**d**) distribution of MRR per unit contact length on the workpiece surface.

Lai et al. [110] studied a wire saw dynamics model and processing mechanism. In Figure 7a, a time curve of the MRR throughout the sawing process is illustrated by the light-colored curve, while the average MRR within each cycle of wire reciprocation is represented by the dark-colored curve. The average MRR exhibits a sharp increase in the initial few hundred seconds, confirming the insufficient sawing force during the initial stage. As the sawing progresses, the average MRR stabilizes, but it rapidly decreases in the final stage. Throughout most of the processing time, the average MRR remains around 0.1 mm^3^/s, showing a consistent trend with the average normal force. Additionally, the MRR exhibits cyclic variations, including instances in which it reaches a minimum value of zero. These fluctuations can be attributed to wire reversal, which causes the absolute wire speed to drop to zero. Figure 7b illustrates the variations in MRR within the wire reciprocation cycle. The MRR immediately increases or decreases when the wire accelerates or decelerates. Stable fluctuations in the MRR are observed when the wire operates at a consistent speed. These fluctuations are solely attributed to the oscillation of the workpiece. The variation in the MRR within one reciprocating cycle is illustrated in Figure 7c. During the oscillation, the MRR reaches its minimum value when the oscillation angle is close to zero, and it attains its maximum value when the workpiece reaches the extreme position. The maximum value within one reciprocating cycle is nearly twice the minimum value. In summary, the MRR in the wire sawing process is unstable overall, as its fluctuations are strongly influenced by both the wire reciprocation motion and workpiece oscillation. According to Chen and Gupta [114], the MRR per unit contact length is influenced by the cutting depth of each abrasive grain during wire saw cutting, which in turn affects the surface quality. By combining the simulated contact length with the actual contact area, the MRR per unit contact length is mapped onto the surface of the workpiece, as shown in Figure 7d.

Dai et al. [90] conducted a study using laser-processed nanoscale diamond tools to investigate the influence of tool groove direction, depth, width, coefficient, and shape on material deformation. The research findings indicate that for silicon workpieces, tools with smaller groove depths, directions, and widths and larger groove coefficients can achieve better ductile cutting and improve the MRR. Chung et al. [138] investigated the distribution of diamond abrasives on the wire for wire sawing processes. The results revealed that a larger spacing between the abrasives leads to a higher MRR. Additionally, this spacing must exceed a critical value to introduce brittle indent cracks, thereby enabling more efficient material removal.

### 3.3. Surface Morphology and Subsurface Damage

Extensive research has been conducted by researchers on the DWS technique. The main characteristics of silicon wafer surface quality include the average surface roughness, surface damage layer thickness, and subsurface cracks [139]. The proportion of brittle and ductile material removal mechanisms, variations in silicon wafer thickness, and wafer strength are also important factors [140,141]. The surface damage layer thickness refers to the depth of the layer that experiences structural alterations and defects during processing, such as sawing or polishing [142]. This layer is characterized by dislocations, microcracks, and other structural deformations. The thickness of the damage layer is crucial because it affects the mechanical strength, electrical performance, and overall reliability of the silicon wafer [143]. Minimizing the surface damage layer thickness is essential to ensuring the production of high-quality and defect-free semiconductor devices. Subsurface cracks are another significant concern in the quality assessment of silicon wafer surfaces. These cracks are located below the surface and can propagate into the bulk material, potentially compromising the mechanical integrity of the wafer. Subsurface cracks can result from various factors, including mechanical stresses, thermal gradients, and material defects. The presence of subsurface cracks significantly reduces the mechanical strength of the wafer and increases the risk of failure during subsequent processing steps or equipment operation [144]. To improve the surface quality of silicon wafers, various optimization strategies are employed in the processes of fabrication. These strategies include optimizing sawing parameters, such as the feed rate, cutting speed, and blade design, to minimize surface roughness and damage [145]. Additionally, the development of advanced polishing techniques and the use of protective coatings can further enhance the surface quality and reduce the occurrence of subsurface cracks [146].

To enhance our understanding of how DWS cutting affects feed force and wafer quality, Costa et al. [113] conducted an experiment in which a single diamond wire was used in a circular configuration. As the wire cutting speed increased, a reduction in the penetration depth of the diamond particles was observed, resulting in a 66% decrease in the feed force. Subsequent analyses indicated that alterations in the feed rate and wire cutting speed exhibited notable effects on the surface roughness parameters Ra and Rq, whereas their influences on Rz were relatively insignificant. Increasing the feed rate led to an increase in the depth of the microcracks, whereas increasing the wire cutting speed resulted in a decrease in microcrack depth. The experimental findings also revealed a significant connection between the surface generated by the saw-cut and the damage occurring beneath it. This correlation indicates that the depth of the microcracks was approximately 1.37 times greater than the Rz value. Moreover, by implementing a combination of reducing the feed rate, decreasing the wire tension, and increasing the wire cutting speed, it was possible to achieve a decrease in the feed force, a smoother surface, and shallower microcracks. In summary, Costa et al.’s research findings highlight the significant influence of cutting parameters such as the wire cutting speed, feed rate, and wire tension on the feed force and silicon wafer quality during the DWS cutting of monocrystalline silicon. By effectively modifying these parameters, it becomes feasible to attain a decrease in the feed force, a smoother surface, and shallower microcracks. Consequently, this enhancement contributes to improving the quality of silicon wafers.

**Figure 8:**
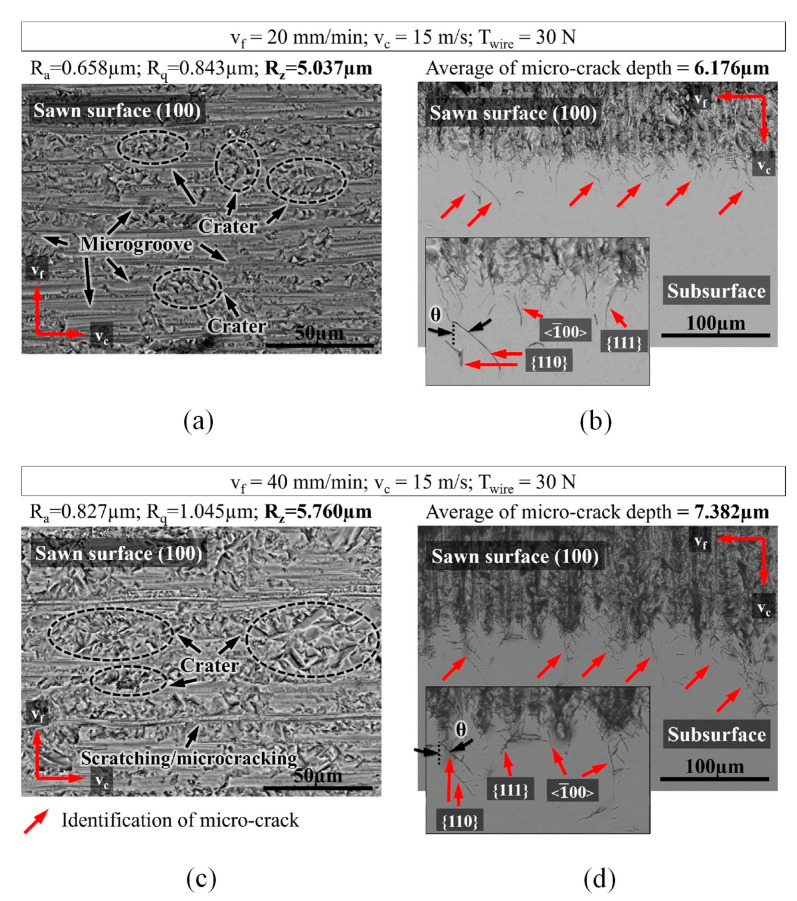
Surface morphology and subsurface damage characteristics in monocrystalline silicon wafer sawing, (**a,c**) shows the sawn surface in the crystallographic plane {100}, (**b**,**d**) location of median microcracks in the subsurface region [113].

A thorough assessment was carried out on the surface topography and subsurface damage characteristics of monocrystalline silicon after sawing, as depicted in Figure 8. Figure 8a,c illustrate the crystal plane {100}. It can be observed that a combination of brittle and ductile cutting exists, along with the presence of pits and microgrooves, which predominantly contribute to variations in the surface roughness, consistent with the values indicated in the SEM images. The primary mechanism for inducing ductile cutting on the {100} silicon plane involves a high-pressure phase transformation [146]. A certain proportion of residual damage is formed underground in the form of median microcracks. Figure 8b,d reveal the presence of predominant median microcracks in the subsurface region, displaying a slight inclination in their preferred propagation direction. In silicon, there are two significant cleavage planes: {111} and {110}. Hence, considering the θ angle, it can be concluded that microcracks predominantly propagate along the preferred crystal plane in the {110} direction on the {110} cleavage plane. Additionally, some microcracks are observed to form along directions on the {111} plane.

Various factors such as material removal mode, processing parameters, and wire saw parameters can affect the surface quality of monocrystalline silicon in the DWS process. These factors, for example, can influence the subsurface microcrack damage depth generated during the process of slicing monocrystalline silicon workpieces. Many studies have been conducted on the subsurface microcrack damage of silicon wafers generated via the DWS of monocrystalline silicon. A predictive model was proposed by Xiao et al. [27] to assess subsurface damage in silicon wafers, taking into account both median and lateral cracks. The effects of the processing parameters on the resulting subsurface damage and surface roughness of the silicon wafers were further investigated. Current research findings generally suggest that when diamond grains scratch or cut monocrystalline silicon, median cracks and radial cracks deviate in the direction of the cutting forces, and the angle of crack deviation significantly affects the depth of subsurface microcrack damage in the crystal. After conducting research on the DWS of sapphire, which is also a hard and brittle material like monocrystalline silicon, Gupta et al. [147] found that the surface roughness and subsurface damage level decreased as the diamond grain size decreased. Additionally, increasing the wire saw speed and improving the uniform distribution of grains on the wire surface led to a continuous reduction in surface roughness and subsurface damage. Kang et al. [148] observed distinct differences in subsurface microcrack depth between the fixed and free abrasive cutting methods when a constant wire speed and feed rate were maintained. The fixed diamond wire exhibited significantly smaller damage depth compared to the free abrasive cutting process.

### 3.4. Summary

The developmental trends of monocrystalline silicon DWS in industrial practice have seen several significant advancements. For instance, the current focus in the industry is on reducing the diameter of the diamond wires to minimize kerf loss, necessitating more precise control over DWS equipment [149]. The wafer dicing industry has also made advancements in manufacturing finer diamond wires. Currently, the use of fine wires is employed in cutting thick wafers to reduce kerf losses, specifically the Si loss equal to the wire diameter. It is anticipated that fine diamond wires with diameters of 50 µm will become mainstream in the near future [150]. As diamond wire diameter significantly decreases, tension also decreases. However, precise tension control is crucial in avoiding sudden wire breakage [151]. Furthermore, in industrial-grade applications, the stability, reliability, and robustness of the DWS process are of paramount importance [152].

DWS is a commonly used machining method for cutting monocrystalline silicon. Its principle involves the use of a wire embedded with diamond particles for cutting. During this process, the wire maintains a constant mechanical speed and applies a uniform force to effectively cut the silicon crystal, facilitating the precise separation of silicon wafers. One key factor in the DWS process is the MRR, which refers to the amount of silicon material removed during cutting. The MRR is mainly influenced by factors such as the cutting speed, feed rate, and wire size. Higher MRR values usually indicate higher levels of production efficiency but may result in greater degrees of surface roughness and subsurface damage. The surface morphology and quality of the cutting surface in DWS are also affected by multiple factors. The size, shape, and distribution of the diamond particles have significant impacts on surface morphology. Smaller and uniformly distributed particles generally provide smoother surfaces. Additionally, the wire’s traverse speed and tension can influence the quality of the cutting surface. Higher traverse speeds and appropriate tension can reduce the impact of cutting forces, thereby reducing surface roughness and subsurface damage. Subsurface damage is an important issue in DWS. During the cutting process, median cracks and radial cracks deflect along the direction of cutting forces, causing small cracks in the subsurface layer of the crystal. The depth and density of these subsurface microcracks are influenced by several factors, including the geometric characteristics of the diamond particles, the cutting parameters, and the crystal structure of the silicon. Typically, smaller diamond particles, lower cutting forces, and higher cutting speeds can reduce the depth of subsurface damage.

In summary, the MRR, surface morphology, and subsurface damage in the morphology of silicon machined via DWS involve the interaction of multiple machining parameters. Understanding the impact of these interactions on cutting quality could help to optimize machining parameters and improve the cutting process to obtain higher-quality monocrystalline silicon wafers. 

## 4. Hybrid Machining

### 4.1. Ultrasonic Vibration-Assisted DWS

**Figure 9:**
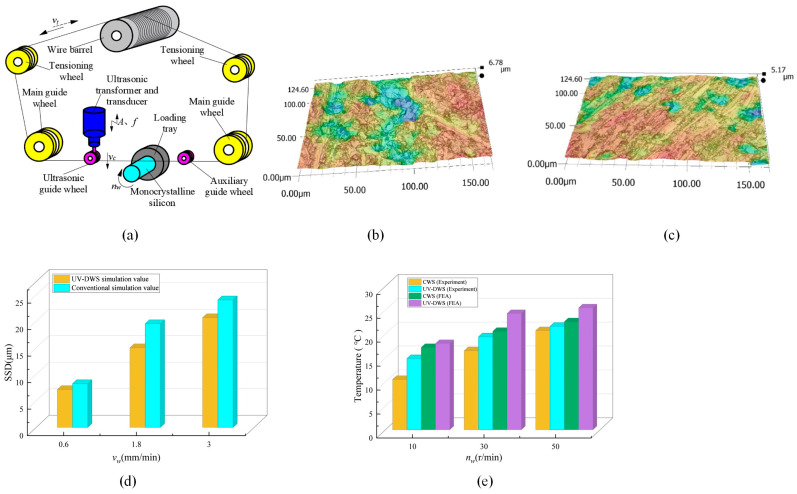
Ultrasonic vibration-assisted DWS: (**a**) principle diagram of UV-DWS [156]; DWS (**b**) and UV-DWS (**c**) wafer surface topography [161]; (**d**) SSD values for different *v_w_* values; (**e**) maximum sawing temperatures at different *n_w_* values.

Ultrasonic vibration-assisted DWS (UV-DWS) is a composite processing technology that combines ultrasonic vibration with fixed abrasive DWS [153]. The ultrasonic vibration-assisted machining process makes it possible to cut a variety of hard materials with conventional diamond [154]. Ultrasonic-assisted vibration has been widely used in numerous manufacturing processes to improve process performance [155]. Figure 9a illustrates the operational concept of UV-DWS in which a monocrystalline silicon cylinder serves as the workpiece. The filament cylinder employs a closed-ring system, comprising a tensioning guide wheel, a leading guide wheel, an ultrasonic guide wheel, and an auxiliary guide wheel to facilitate the DWS process. When the wire saw is cutting, the wire saw rotates with the motor, driving the wire saw to make reciprocating cutting motion with a speed *v_t_* on the wire saw. At the same time, the wire saw is driven by the frame to feed downward at a speed *v_c_*, and the loading tray securely holds the workpiece and enables it to rotate uniformly around its own axis at the specified speed of *n_w_*. Ultrasonic excitation with an amplitude *A* and as frequency *f* is applied along the ultrasonic guide wheel feeding direction to make the wire saw perform ultrasonic compound processing [156].

UV-DWS is widely used as an advanced machining technique for the processing of hard and brittle materials [157]. The utilization of ultrasonic vibration in machining technology offers the benefit of creating intermittent contact between the workpiece and the tool [158]. Wang et al. [156] showed experimentally that UV-DWS had a sawing force that was on average 38% lower sawing force, lower surface roughness values, and produced an improved surface topography when compared to conventional DWS. Subsurface damage caused by machining is a major obstacle to the widespread application of hard and brittle materials [159]. Wang et al. [160] proposed that SSD in DWS silicon wafers has a great influence on the subsequent processing process and fracture strength of silicon wafers. After employing both methods, the surface morphology of the sawed silicon wafers was examined by Wang et al. [161] using optical microscopy. Under the same cutting conditions, the number of craters produced by UV-DWS on the silicon wafer surface was less than that of DWS, and the craters were small and shallow. The surface morphology is shown in Figure 9b,c. The average reduction in SSD for UV-DWS compared with DWS is 23.75%. Different sawing parameters have different effects on the SSD. Figure 9d shows the SSD for different wire saw feed rates *v_w_* for both methods. In addition, Wang et al. [161] conducted a comparative study of the surface roughness of workpieces processed via DWS and UV-DWS. The experimental results showed that the surface roughness of the workpiece machined via UV-DWS was 4.3~29.7% lower than the one machined via DWS. Wang et al. [140] investigated the effects of different sawing parameters and ultrasonic vibrations on the sawing temperature when performing DWS on monocrystalline silicon. Under the same sawing parameters, the sawing temperature of UV-DWS is about 1.5 °C higher than that of DWS. The maximum sawing temperature of DWS was 27.9 °C and the maximum sawing temperature of UV-DWS was 29.9 °C, indicating that ultrasonic vibration does not significantly change the sawing temperature. Figure 9e shows the maximum sawing temperatures at different *n_w_* values.

### 4.2. Electrical Discharge-Assisted DWS

**Figure 10:**
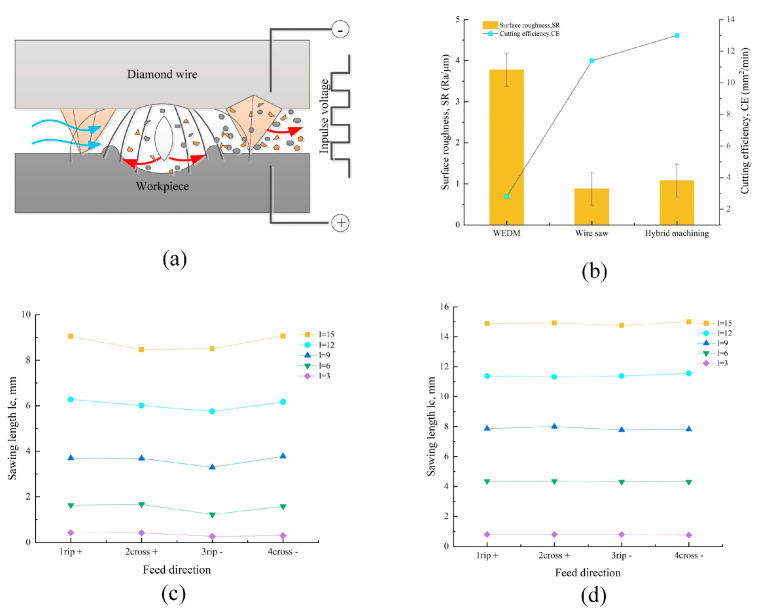
Electrical discharge-assisted DWS: (**a**) principle diagram of ED-DWS [125]; (**b**) surface roughness and cutting efficiency of three machining methods; machining accuracy of DWS (**c**) and ED-DWS (**d**).

Electrical discharge vibration-assisted DWS (ED-DWS) is a highly promising new technology for silicon slicing [162]. The combination of electric discharge machining (EDM) [163,164,165,166] and the DWS method offers several advantages over the current DWS method, including improved sawing efficiency, reduced kerf loss, minimized line marks, and enhanced surface uniformity [167,168,169]. ED-DWS is accomplished by connecting the workpiece to the positive terminal of a high-frequency discharge power supply while the diamond wire is connected to the negative terminal, creating an open-circuit voltage between them. This setup generates an electrical discharge effect that aids in material removal during the cutting process. Figure 10a shows the principle diagram of ED-DWS [125].

Wu et al. [170] proposed a DWS process combining EDM cutting and fixed abrasives in the semiconductor field which has the advantages in terms of reducing silicon surface scratches and tool wear. The cutting efficiency and surface roughness of the three methods were compared. As can be seen in Figure 10b, compared with EDM wire cutting, combining wire sawing with compound machining can lead to a significant reduction in surface roughness, decreasing it from 3.8 μm to approximately 1 μm. Additionally, this combined approach results in a substantial improvement in cutting efficiency, increasing it from 8.4 mm^2^/min to around 13 mm^2^/min. The greatest cutting efficiency was achieved via composite machining. Qiu et al. [171] designed a sawing test for longitudinal and cross-cutting with different feed directions to compare the kerf length of DWS and ED-DWS. The kerf length was used as the machining accuracy index. The results are shown in Figure 10c which shows that the machining accuracy of DWS is 1X+ > 4Y-> 2Y+ > 3X-, and there is no significant difference between longitudinal and crosscutting in ED-DWS. The machining accuracy of ED-DWS is significantly better than that of DWS for both longitudinal and transverse cuts, as shown in Figure 10d. Qiu et al. [172] proposed an environmentally improved method for diamond wire EDM sawing under electroplating liquid cooling conditions. The results showed that the machining accuracy using bath cooling was superior to that of jet cooling for both DWS and ED-DWS. In terms of tension, the stability and envelope values of the tension in ED-DWS with both cooling methods surpass those of DWS, indicating improved machining conditions in ED-DWS [173]. In terms of tension, the stability and envelope values of the tension in ED-DWS with both cooling methods surpass those of DWS, indicating improved machining conditions in ED-DWS. 

### 4.3. Electrochemical -Assisted DWS

Electrochemical-assisted DWS (EC-DWS) is a new method for machining high-hardness and brittle materials. The generation of a high temperature via electrochemical discharge promotes the movement of the diamond wire, resulting in material flaking and consequently enhancing the MRR [174]. Wire EDM is a widely used non-traditional machining process that is suitable for hard and brittle materials. It is increasingly significant due to its non-contact machining approach, which offers advantages such as low surface damage and high machining efficiency [175,176]. The EC-DWS process is a combination of EDM and electrochemical machining [177]. By applying a DC voltage to both the wire (serving as the negative electrode) and the auxiliary electrode (acting as the positive electrode), the electrochemical reaction initiates, forming a hydrogen film that envelops the wire. This film serves as insulation against the electrolyte. When the DC voltage surpasses the breakdown threshold, an electric spark emerges within the gas film surrounding the wire [178]. Wang et al. [179] conducted a detailed study on the surface integrity of high-hardness, brittle materials cut via electrochemical discharge-assisted DWS. The combination of electrochemical discharge and DWS was observed to enhance the surface roughness. Initially, the surface roughness increased and then decreased with the increase in DC voltage.

**Figure 11:**
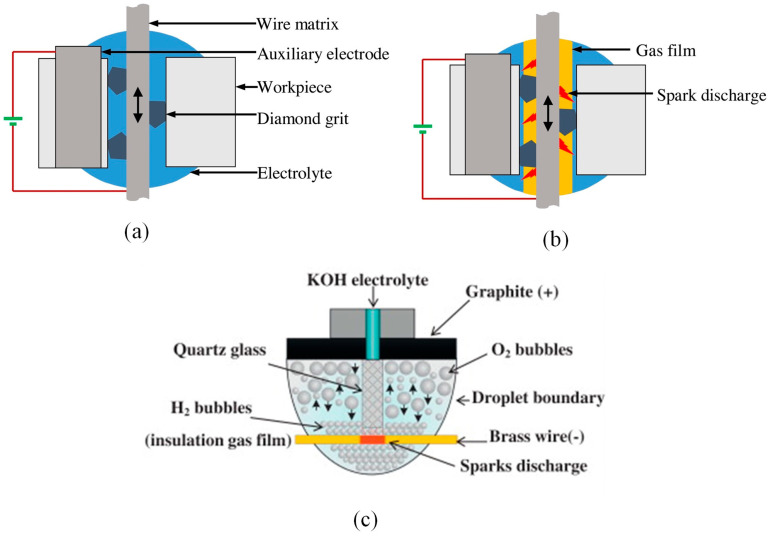
Electrochemical-assisted DWS (**a**) without the application of DC voltage [180]; (**b**) with the application of DC voltage higher than the critical value [180]; (**c**) illustration of H_2_ and O_2_ bubbles within KOH droplets during the EC-DWS process [181].

Figure 11a illustrates the principle of spark discharge generated by the EC-DWS process during diamond wire cutting. In this process, the diamond wire is connected to the negative terminal, while the L-shaped auxiliary electrode is connected to the positive terminal of a DC power supply. Figure 11b depicts a process in which the electrolyte is sprayed into the cutting gap through a nozzle, establishing an electrical circuit between the diamond wire and the auxiliary electrode. Initially, at a lower applied DC voltage, an electrochemical reaction takes place on the diamond wire, resulting in the generation of hydrogen gas. With an increase in the voltage, the production of hydrogen gas intensifies, eventually forming a protective gas film surrounding the diamond wire. This film, being extremely thin, insulates the diamond wire from the electrolyte. Consequently, the majority of the applied voltage from the DC power supply is directed toward the diamond wire and the electrolyte. Once the voltage surpasses a critical value, spark discharge occurs within the gas film. The material removal process is enhanced by the instantaneous temperature increase and the reduction in the strength of the workpiece material caused by the spark discharge. This phenomenon promotes the movement of the diamond wire, leading to improved material removal [180]. In EC-DWS techniques, electrode wires made of brass, copper, and chemically reactive graphite auxiliary electrodes are commonly used. The electrolyte, which can be KOH or NaOH, is supplied continuously in a coaxial manner with the wire electrolyte. By applying a high potential difference between the two electrodes, droplets are formed. Within these droplets, the auxiliary electrode generates oxygen gas bubbles, while the electrode wire generates hydrogen gas bubbles, as depicted in Figure 11c. The formation of a stable insulating gas film, resulting from the accumulation of bubbles, initiates sparks and facilitates material removal [181].

### 4.4. Summary

In addition to the methods for the composite processing of monocrystalline silicon, non-silicon-based materials are used as substitutes for silicon-based materials in specific situations or applications. Cadmium telluride photovoltaic (CdTe PV) technology has been developing rapidly and contributes greatly to global non-silicon-based PV technology [182]. Aghaei et al. [183] compared thin-film solar cells such as CdTe with conventional silicon solar cells and showed that thin-film solar cells such as CdTe were more economical. Petter Jelle et al. [184] stated that the typical efficiencies of monocrystalline cells were 16–24%, and copper indium diselenide (CIS) and copper indium gallium selenide (CIGS) cells are the most efficient thin film cells available, with typical cell efficiencies of 11–18.7%. Muteri et al. [185] analyzed the environmental impact of the life cycle of PV technologies from the first generation (conventional silicon-based) to the third generation (innovative non-silicon-based). The second and third generations require less energy in the manufacturing process than silicon processing and in most cases, have lower environmental impacts. In the field of non-silicon solar PV cells, there has been a significant increase in the production of dye-sensitized solar cells, increasing the likelihood of the emergence of highly effective and efficient solar cells [186]. The use of carbon nanotubes for chip fabrication could solve the problem that silicon technology has ceased shrinking the chip size to meet the predictions of Moore’s law [187]. Choi et al. [188] proposed type IV-B metal oxides, especially HfO_2_, ZrO_2_, and TiO_2_, metal oxides that were superior to SiO_2_ in terms of their higher dielectric constants and were attracting attention due to their potential as gate dielectric materials to replace SiO_2_ and have attracted much attention.

Composite methods for processing monocrystalline silicon utilize a combination of ultrasonic vibration, EDM, electrochemical-assisted DWS, and DWS. These auxiliary methods can improve the shortcomings of the DWS of monocrystalline silicon. The UV-DWS of monocrystalline silicon is accomplished by applying ultrasonic excitation along the feed direction using an ultrasonic guide wheel for ultrasonic composite processing. Compared with conventional DWS, the cutting force is reduced, the surface roughness is lower, and the surface quality is improved, and ultrasonic vibration has little effect on the cutting temperature. The ED-DWS process creates an open circuit voltage between the workpiece and the diamond wire to produce an electrical discharge effect to assist in removing the material. This method uses the heat generated by the electrical discharge to soften the material and facilitate processing. It is found that the machining accuracy of ED-DWS is higher than that of DWS in terms of cutting direction. The quality of the cut monocrystalline silicon can also be improved by changing the ambient cooling conditions of ED-DWS. EC-DWS is when voltage is applied to both the diamond wire and the electrolyte. The voltage exceeds a critical value, the diamond wire surface is discharged, and sparks are generated. The elevated temperature generated by the spark diminishes the strength of the workpiece, thus facilitating the removal of the workpiece material by the diamond wire.

In conclusion, cutting monocrystalline silicon using the composite method is better than diamond cutting monocrystalline silicon, as measured from multiple perspectives, such as cutting efficiency and surface roughness. Even the use of ultrasonic vibration reduces the depth of the subsurface damage of monocrystalline silicon. The processing quality of monocrystalline silicon can be improved. Table 3 provides a summary of hybrid machining-assisted types of precision-machining DWS.

## 5. Outlooks

DWS is a prominent method for precision machining monocrystalline silicon, and it holds great potential for further advancements and improvements. Looking ahead, several key areas offer opportunities for progress and innovation in this field:

(1) The development of advanced modeling and simulation techniques can aid in the optimization of the cutting process. By utilizing computational models, the complex interrelationships among the cutting tool, the workpiece, and the process parameters can be analyzed [27,110]. Multiple research methods could be combined, such as mathematical modeling with MD, MD with an FEM simulation, or a combination of these three methods. These models can provide insights into material removal mechanisms, stress distributions, and temperature profiles, enabling the prediction and control of surface quality and subsurface damage.
(2) Optimizing the cutting parameters is crucial for achieving greater precision and surface quality. By systematically studying the effects of these process parameters, it becomes possible to understand their complex interplay and identify the optimal settings. Adjusting the wire tension can influence the stability and vibration characteristics of the diamond wire, which in turn affect the cutting process. By understanding the complex interplay between these parameters, it is possible to identify optimal settings that minimize surface roughness and subsurface damage while maximizing productivity.
(3) Combining UV-DWS, ED-DWS, and EC-DWS methods with DWS can enhance the processing of monocrystalline silicon. By combining these methods, the cutting process can be optimized to achieve greater efficiency, better surface quality, and precise control over the cutting parameters. This combination of techniques holds great potential for advancing the DWS of monocrystalline silicon and similar materials. By combining these methods, the cutting process can be optimized to achieve a higher level of efficiency, better surface quality, and precise control over the cutting parameters. Process methods such as laser ultrasound-assisted DWS or a combination of other auxiliary methods may also be introduced in the future to further improve processing quality [189,190]. Non-silicon-based technologies have gained attention due to their unique properties and potential advantages over traditional silicon-based approaches. These technologies offer different characteristics and performance capabilities that may be advantageous in terms of flexibility, energy efficiency, or higher operating frequencies.
(4) Artificial intelligence (AI) technology is growing in various industries. The integration of internal monitoring and feedback systems can enable real-time process control and quality assurance. Machine learning can enable the real-time monitoring of key process parameters and provide feedback for adaptive control [191,192,193]. By incorporating sensors and measurement techniques [194], it becomes possible to monitor key parameters such as the cutting force, temperature, and surface roughness during the cutting process [195,196]. This information can be used to adjust cutting parameters on the fly and ensure consistent and high-quality results.

## Figures and Tables

**Table 1:** Summary of DWS precision machining models and simulation types.

Types of Models and Simulations	Authors, Year	Purpose	Findings	Remarks
Mathematical model	Li et al., 2019 [42]	Based on indentation fracture mechanics, a mathematical model of the influence of process parameters and wire saw parameters was developed.	The areas of brittle cracks produced by the abrasive can affect the surface morphology of the wafer.	Larger feed rates and line speeds increase the cutting efficiency and make it easier to obtain a surface of brittle excised material.
	Wu et al., 2013 [43]	The effects of crystal defects on the cutting performance of polysilicon were investigated.	At the critical cutting depth of the ductile-brittle transition of the material, there was a significant variation within the particles.	A higher dislocation density is associated with greater fracture toughness and larger critical depth of cut.
	Yin et al., 2021 [44]	A mathematical model of DWS was established, and the sawing process was numerically calculated.	The critical ratio of the workpiece feed speed to the saw wire motion speed was obtained with a combination of different parameters.	Increasing the speed of the saw wire movement or decreasing the feed speed of the workpiece is more beneficial to achieving material removal.
MD model	Liu et al., 2022 [74]	The atomic structures of orthocrystalline silicon crystals and silicon nanowires were compared.	Strain rate sensitivities and critical strain rates were obtained for both structures using a rate reactivity model.	A calculation of both rates revealed that the additional surface of THE SiNW reduced the sensitivity of the strain rate.
	Olufayo et al., 2013 [89]	MD simulation for the atomic visualization of plastic material flow at the tool-workpiece interface during orthogonal cutting.	The simulated MD force and temperature outputs were evaluated to obtain the accuracy of the model.	The MD method can be used to study the atomic reactions on the tool/workpiece surface, revealing the ductile transition response of the nanoprocess.
	Dai et al., 2017 [90]	MD simulation of the cutting of monocrystalline silicon with laser-fabricated, nanostructured diamond tools.	The effects of different trench orientations, depths, widths, factors, and shapes on the nanoscale cutting process were investigated.	Groove orientation has a significant effect on the nanoscale cutting process, and cutting with V-shaped grooves can improve material removal.
FEM	Wei et al., 2018 [52]	The thickness and stress strength factors of monocrystalline silicon, as well as the crack extension angle, were studied via MD simulation and FEM, respectively.	The thickness and stress strength factors, as well as the crack extension angle, were obtained via MD simulation and FEM, respectively.	The critical stress strength factors and crack extension angles are clearly dependent on the chiral angle, thickness, and loading angle of the monocrystalline silicon plate.
	Zhang et al., 2014 [53]	Anisotropic effects in silicon were evaluated using stiffness and flexibility coefficient matrixes.	Proper crystal orientation can improve performance and reduce mechanical bending stress.	For monocrystalline silicon, heat deformation can be approximated by using the isotropic constant Poisson’s ratio.
	Skalka et al., 2021 [91]	An FE simulation and optimization procedures were used to determine the cohesive energy density of monocrystalline silicon.	The adhesion energy density was evaluated and the material toughness was determined.	The reliability of the model originates from the comparison of the numerical simulation results with the measured data.

**Table 2:** Summary of the effect of parameters on the stability of the sawing process.

Parameters	Authors, Year	Purpose	Findings	Remarks
Tension	Albrecht and Möhr-ing, 2018 [107]	The effect on the stability of the sawing process was investigated experimentally and by simulation.	At higher tensions (350 MPa and 400 MPa), saw blade displacement remained essentially the same, while higher tensions resulted in reduced displacement.	Adjusting the saw blade parameter tension during the cutting process does not affect the processing time.
Cutting speed, feed rate, and wire tension	Costa et al., 2020 [108]	To investigate the effect of DWS on the surface integrity of monocrystalline silicon.	For two wire tensions (Twire) = 30 N, the Sa value increased significantly when compared with the specimens sawn using Twire = 20 N.	The most suitable set of cutting parameters is the lowest feed rate and wire tension and the highest wire cutting speed.
Stiffness of wire web, tension, fluctuation of wire, and reciprocating period	Qiu et al., 2021 [109]	To study the factors affecting the machining accuracy of circular diamond rope saws and their mechanisms.	The roughness value of endless wire sawing was Ra = 1.6 µm and that of reciprocating sawing was Ra = 1.254 µm.	Stable tension corresponds to better machining accuracy.
Wire speed, feed rate, rocking angle, preload force, and guide roller distance	Lai et al., 2023 [110]	To analyze the effect of machining parameters on sawing force, contact length, and MRR.	Workpiece rocking reduces contact length, with a maximum contact length of about 20% of the workpiece diameter during sawing.	Feed speed, maximum wire feed speed, maximum swing angle and preload force all affect the range of MRR fluctuations.
Reciprocating period and sawing arc length	Dong et al., 2021 [111]	A reciprocating oscillating motion pattern was introduced in a cutting frame saw to study the cutting performance of sawing.	The depth of the cut and the distribution of the sawing force depend on the position of the saw blade on the saw surface.	The effect of sawing conditions on sawing force is related to the depth of cut of the cutter head.

**Table 3:** Summary of hybrid types of machining -assisted precision-machining DWS.

Types of Hybrid Machining	Authors, Year	Purpose	Findings	Remarks
UV-DWS	Wang et al., 2022 [156]	Conducting theoretical research on the cutting force of UV-DWS based on abrasive wear.	A theoretical model of UV-DWS force from single to multiple abrasive grains was developed.	Compared with DWS, UV-DWS can reduce the sawing force and improve the flatness of the workpiece.
	Wang et al., 2023 [140]	UV-DWS of monocrystalline silicon SSD.	A mathematical model of UV-DWS damage to silicon wafers was developed, and the law of SSD was analyzed.	The UV-DWS monocrystalline wire silicon model verifies that the SSD varies with different sawing parameters.
	Wang et al., 2019 [161]	Modeling and validation of UV-DWS cutting force based on impact loading.	The validity of the impact loading was demonstrated using the UV-DWS.	The surface quality of UV-DWS is better than that of DWS.
ED-DWS	Wu et al., 2018 [170]	A pilot study of EDM wire cutting and fixed abrasive wire saw compound machining was conducted.	A composite machining method combining EDM wire cutting and fixed abrasive DWS together was studied.	Compared with fixed abrasive DWS, the hybrid processing method reduces silicon surface scratches.
	Qiu et al., 2023 [171]	The machining accuracy of DWS and ED-DWS in longitudinal and transverse sawing was compared.	Better machining accuracy and surface quality are achieved with ED-DWS under bath cooling than under jet cooling.	ED-DWS outperforms DWS in terms of machining accuracy and cutting efficiency.
	Qiu et al., 2023 [172]	An environmentally improved method of ED-DWS under plating solution cooling conditions was proposed.	Its advantages were compared with those of jet cooling through a series of sawing tests.	The roughness of bath cooling is better than jet cooling, but the fluidity becomes worse and chip removal becomes difficult.
EC-DWS	Wang et al., 2017 [179]	Electrochemical discharge-assisted DWS cutting of hard and brittle materials for surface integrity.	Based on the experimental results, each element of the machined surface was analyzed.	The combination of electrochemical discharge and DWS can improve surface roughness.

## Data Availability

Not applicable.
